# Novel Functions and Regulation of Cryptic Cellobiose Operons in *Escherichia coli*

**DOI:** 10.1371/journal.pone.0131928

**Published:** 2015-06-29

**Authors:** Vinuselvi Parisutham, Sung Kuk Lee

**Affiliations:** 1 School of Life Sciences, Ulsan National Institute of Science and Technology (UNIST), Ulsan, Republic of Korea; 2 School of Energy and Chemical Engineering, Ulsan National Institute of Science and Technology (UNIST), Ulsan, Republic of Korea; University of Houston, UNITED STATES

## Abstract

Presence of cellobiose as a sole carbon source induces mutations in the *chb* and *asc* operons of *Escherichia coli* and allows it to grow on cellobiose. We previously engineered these two operons with synthetic constitutive promoters and achieved efficient cellobiose metabolism through adaptive evolution. In this study, we characterized two mutations observed in the efficient cellobiose metabolizing strain: duplication of RBS of *ascB* gene, (β-glucosidase of *asc* operon) and nonsense mutation in *yebK*, (an uncharacterized transcription factor). Mutations in *yebK* play a dominant role by modulating the length of lag phase, relative to the growth rate of the strain when transferred from a rich medium to minimal cellobiose medium. Mutations in *ascB*, on the other hand, are specific for cellobiose and help in enhancing the specific growth rate. Taken together, our results show that *ascB* of the *asc* operon is controlled by an internal putative promoter in addition to the native cryptic promoter, and the transcription factor *yebK* helps to remodel the host physiology for cellobiose metabolism. While previous studies characterized the stress-induced mutations that allowed growth on cellobiose, here, we characterize the adaptation-induced mutations that help in enhancing cellobiose metabolic ability. This study will shed new light on the regulatory changes and factors that are needed for the functional coupling of the host physiology to the activated cryptic cellobiose metabolism.

## Introduction

There are at least four operons (*chb*, *asc*, *bgl*, and *bgc*) [1–4] for the metabolism of β-glucoside sugars such as arbutin, salicin, and cellobiose in *Escherichia coli*. For activation these operons require stringent selection pressure with a particular β-glucoside as sole carbon source in minimal medium for several days to months; hence, they are considered cryptic. Mutations in the regulatory proteins such as ChbR and BglG, or in the promoters, help activate the respective operons for the metabolism of a particular β-glucoside [1,4]. The *bgl* operon is involved in the metabolism of aryl β-glucosides such as salicin and arbutin [1]. In nature, the *chb* operon is not a cryptic operon and is induced by its native substrate, chitobiose. However, mutations in the regulatory proteins (NagC and ChbR) of the *chb* operon allow *E*. *coli* to grow on cellobiose [5]. For the *asc* operon, even with the inactivation of the repressor protein, AscG, or replacing the cryptic promoter with constitutive promoter [6], the resulting strains do not gain an efficient cellobiose metabolizing phenotype but can transport and cleave *p*-nitrophenyl-β-glucopyranoside (a synthetic analog of cellobiose) [3,6]. Sequence similarity suggests that the *asc* operon is probably a duplication of the *bgl* operon of *E*. *coli* [3].

Previous studies for understanding the cryptic cellobiose metabolism were based on characterizing stress-directed mutations in the *chb* and *asc* operons [4]. However, these studies could not establish host factors (other than the genes of the *chb*/*asc* operons) that control the cryptic cellobiose metabolism [4]. We have previously engineered a genetically modified *E*. *coli* strain expressing both *asc* and *chb* operons constitutively (hereafter referred to as OSS, original synthetic strain). Thus, the strain OSS expresses eight genes (*chbB*, *chbC*, *chbA*, *chbR*, *chbF*, *chbG*, *ascF* and *ascG*) constitutively, of which *chbBCA* and *ascF* encode the PTS-mediated transporter, whereas *chbF* and *ascB* encode for phospho-β-glucosidase. These strains were adapted on cellobiose minimal medium for 30 days to achieve efficient cellobiose metabolism and the resulting strain was named ESS (evolved synthetic strain) [6].

Here, we describe the functional benefits of the activated *chb* and *asc* operons and decipher the regulatory changes that occurred during adaptation on cellobiose minimal medium. In bacteria, the transcriptional regulatory network plays a significant role in helping bacteria to adapt to the nutrients in the medium. Detailed knowledge of the endogenous regulatory network that shows differential response to the activated cryptic genes is thus important to rewire metabolic pathways for efficient cellobiose metabolism. While previous studies characterized the stress-induced mutations that allowed growth on cellobiose [4], here, we characterize the adaptation-induced mutations that help in enhancing cellobiose metabolic ability. This study will shed new light on the regulatory changes and factors that are needed for the functional coupling of the host physiology to the activated cryptic cellobiose metabolism.

## Materials and Methods

### Bacterial strains and media composition

All strains and plasmids used in this study are listed in Table 1 [6]. Bacteria were cultured at 37°C in Luria Bertani broth (LB) or minimal medium supplemented with cellobiose. Strains carrying temperature-sensitive plasmids were grown at 30°C. Media were supplemented with suitable antibiotics (30 μg chloramphenicol/mL and 100 μg ampicillin /mL). For long-term storage, cells were maintained as 20% glycerol stocks at –80°C.

**Table 1:** Bacterial strains and plasmids used in this study.

Strains/plasmids	Description/genotype	Reference/source
*Strains*		
*E*. *coli* MG1655	Wild type	[23]
OSS	MG1655 with *chb* and *asc* operon promoters replaced with CP12	[6]
ESS	OSS adapted in cellobiose for 30 days	[6]
OSS-yebK*	OSS with nonsense mutation in *yebK*	This study
OSS-ascB*	OSS with 10-*nt* inserted in the RBS region of *ascB*	This study
OSS-yebK*/ascB*	OSS with both mutations in ESS	This study
MG1655/ΔP_asc_-*ascB*::frt	MG1655 with P*_ascG_* to *ascB* deleted	This study
MG1655/Δ*chbB*- *chbF*::frt	MG1655 with *chbB* to *chbF* deleted	This study
OSS-Δ*eda*::*frt*	OSS with *eda* gene deleted	This study
OSS-Δ*edd*::*frt*	OSS with *edd* gene deleted	This study
OSS-yebK*/Δ*edd*::*frt*	OSS with nonsense mutation in *yebK* and *edd*gene deleted	This study
OSS- P2E5	OSS with RBS of *ascB* mutated through MAGE	This study
*Plasmids*		
pSIM5	λ-Red recombinase expression plasmid and temperature-sensitive replication	[24]
pCP20	Yeast FLP recombinase gene controlled by CI repressor and temperature sensitive replication.	[25]
pKD13	Template plasmid for gene disruption. The kanamycin resistance gene is flanked by FRT sites. *ori*R6K requiring the *pir*+ *E*. *coli*.	[7]
pKD13-SacB	Modified form of pKD13 with kanamycin gene co-expressed with *sacB* gene	This study
pET31b+*yebK*-6his	pET31b+ plasmid with *yebK*-6 His epitope tag	This study
pET31b+yebK*-6his	pET31b+ plasmid with yebK*-6 His epitope tag	This study
pProbe-NT'	pBBR1 ori., Km^R^	[26]
pProbe-A5	pProbe-NT' plasmid containing the sequence + 391 *nt* from the start codon of *ascF* till the end of *ascB* cloned into the *EcoR*I, *Kpn*I site	This study

M9 minimal medium, supplemented with 2 mM MgSO_4_, 0.1 mM CaCl_2_, and 4 g sugar/L, was used to characterize the cell growth rate of the modified strains. In these tests, overnight cultures grown in LB were collected, washed once with M9 salts and suspended to a final OD of 0.05 in 50 mL of M9 medium supplemented with the test sugar in a 250-mL flask, and the cultures were incubated at 37°C with rotation at 200 rpm. Cell growth was monitored by measuring the optical density at 600 nm (OD_600_) every 3 hours with a Biochrom Libra S22 spectrophotometer.

### Whole genome re-sequencing

Genomic DNA was isolated from both OSS and ESS using GeneAll DNA isolation kit. Library construction and DNA sequencing was performed by Macrogen Company (Korea) on an illumina Hiseq2000 platform. *E*. *coli* K12 MG1655 (NC_00913.2) sequence was used as a reference sequence.

### Strain construction

Gene deletion was performed using the λ-Red recombination system, as described previously [7]. Briefly, the kanamycin cassette of pKD13 was amplified using the deletion primers containing 50 *nt* homology to the target genes. Cells carrying the λ -Red system, under the control of the P_L_ promoter (pSIM5), were induced at 42°C for 15 minutes, made electro-competent, and then transformed with the PCR product. Transformant colonies carrying the desired modification were directly selected on LB agar plates supplemented with kanamycin. The kanamycin cassette was then cured using the FLP recombinase expressed from pCP20 plasmid. Genomic DNA was isolated from the transformants, and the target region was PCR amplified and sequenced to confirm site-specific insertion. The primers used for strain construction are listed in Table 2.

**Table 2:** Primers used in the study. Sequence in red indicates the duplicated nuclotide observed in strain ESS.

Primer Name	Sequence
*chbB*_RBS	AACAAAACAGATAAATGTGTTTCTTTTCCATAAAACTGCCCTNNNNNNCGATTATCTGTCAGCCAGACACTCCGCAAGCCTTAACCTGCT
*chbC*_RBS	CCTTTTCAAGCGATGCAATAACATTACTCATAGAAAAATACCNNNNNNAACCGCAATTTAAATATTGCGGTATTGATTTATGAAATAACT
*chbA*_RBS	CGTTTGCGTATCGGGAATGTTATCGAGATCCATCATACATCGNNNNNNTCTTTTCTTACCGGCACGATTACCCGTACCGGCATCGATTAA
*chbF*_RBS	CACCAATAGTGACGACTTTTAATTTCTGGCTCATAATTTCTCNNNNNNGTACAGAATACTGATATCTGGCATATCTGCCCCCCCGGACAT
*ascF*_RBS	ACTAGTCGGCCAAAATGATATAATACCTGAGTACTGTTCACANNNNNNACAGCTATGGCCAAAAATTATGCGGCGCTGGCACGCTCGGTG
*ascB*_RBS	TCAGAGCGTACAACCGACCGTCGCCAAAGAAGTAAGTCTTAANNNNNNATGAAAATGTCAGTATTTCCAGAAAGTTTTTTATGGGGCGGC
*ascB*_ESS_RBS	TACAACCGACCGTCGCCAAAGAAGTAAGTCTTAATT**GAGGATGAAA**GAGGATGAAAATGTCAGTATTTCCAGAAAGTTTTTTATGGGGCG
CP_Direct	CATATACAAGTTTATTCTTGACACTAGTCGGCCAANNNNNNNNNNTACCTGAGTACTGTTCACACAGGAAACAGCTATG
CP_Complement	CATAGCTGTTTCCTGTGTGAACAGTACTCAGGTANNNNNNNNNNTTGGCCGACTAGTGTCAAGAATAAACTTGTATATG
*yebK*_FP	TTTCTTTCAGTGCGGAAATCGTCATTACCCGTGAGTCTCTTTACATCATGGTGTAGGCTGGAGCTGCTTCG
*yebK*_RP	GTATAAGATTAGGACAGTGACAGTCGTTTTTAGCGATCGTCACTTAAATTATTCCGGGGATCCGTCGACC`
*eda*_FP	GCCCGATCCTCGATCGGGCATTTTGACTTTTACAGCTTAGCGCCTTCTACGTGTAGGCTGGAGCTGCTTCG
*eda*_RP	TCACTTTTTAAGACGACAAATTTGTAATCAGGCGAGAGAAAACTCTGATGATTCCGGGGATCCGTCGACC
*edd* FP	TTCTCTCGCCTGATTACAAATTTGTCGTCTTAAAAAGTGATACAGGTTGCGTGTAGGCTGGAGCTGCTTCG
*edd* RP	CGCGTTGTGAATCATCCTGCTCTGACAACTCAATTTCAGGAGCCTTTATGATTCCGGGGATCCGTCGACC
*asc_*del_FP	GAAACCCCGGCGCGCTTCGCCACTTCCAGCATCGTCGTCATCATTTTCATGTGTAGGCTGGAGCTGCTTCG
*asc_*del_RP	GGCGTTCACGCCGCATCCGGCACTGTTACCTACTCTAAATCTTCCCCATTATTCCGGGGATCCGTCGACC
*chb_*del_FP	ATGGAAAAGAAACACATTTATCTGTTTTGTTCTGCGGGCATGTCTACCTCATTCCGGGGATCCGTCGACC
*chb_*del_RP	ATCAGTAAGCGTTCCATAATCAGCCTCGGTTAATGTGCTTTTTTAAGCTCGTGTAGGCTGGAGCTGCTTCG
*ascB*_RT_FP	AATAAATCATAA TTTTTATGGGGCGGCGCGCT
*ascB*_RT_RP	AATAAATCATAA CGTCATCTCGCAACTGAAAACG
*ascF*_RT_FP	AATAAATCATAA TAAAAACCATCCCCGGCGTG
*ascF*_RT_RP	AATAAATCATAA ACGGGCTGTGCGGGCTGCAT
*yebK*_RT_FP	AATAAATCATAA GTCTCAGCTGGAACATTTGAGC
*yebK*_RT_RP	AATAAATCATAA TGCGACAGAAACGATTCACCG
16S_RT_FP	AATAAATCATAA TCTTGCCATCGGATGTGCCC
16S_RT_RP	AATAAATCATAA TGGACCGTGTCTCAGTTCCA
*ascF*-5'-GSP2	TGACGACTTCCTGAAAGGCTTGTGA
*ascB*-5'-GSP2	AAAGCAGGTTCTGGCGTAGCGGCT
Q_T_	CCAGTGAGCAGAGTGACGAGGACTCGAGCTCAAGCTTTTTTTTTTTTTTTTTTTTTTT
Q_0_	CCAGTGAGCAGAGTGACG
Q_I_	GAGGACTCGAGCTCAAGC
YebK_EMSA_FP	GGGTAAAAATCTGACACTGATCATG
YebK_EMSA_RP	TCCGCACTGAAAGAAATCGAAATGC

The underlined sequence indicate the restriction site used for cloning. The randomized RBS sequence is indicated as NNNNNN.

OSS-yebK* was constructed using a scar-less deletion method. The kanamycin cassette in the pKD13 plasmid was modified to include the *sacB* gene. The *kan*-*sacB* cassette was amplified with primers designed to have 50 *nt* homology to the target gene and gene deletion was performed using the λ-Red recombination system as described above. Sucrose sensitive strains were then selected by negative screening on LB sucrose plates (containing no salts). For yebK*, the *yebK* gene was amplified from ESS and transformed to the sucrose sensitive OSS-Δ*yebK*::*kan*-*sacB* strain essentially as described above. The transformants were selected on LB-sucrose plates and confirmed by DNA sequencing.

OSS-ascB* strains were constructed by single stranded oligo-mediated recombineering. Oligos were designed for the insertion of ten nucleotides that were found duplicated in the ESS strains. Genome engineering was performed for four cycles using 2 μM oligo per cycle. Ninety-six colonies were randomly picked and screened on minimal cellobiose medium to identify clones with 10-*nt* inserted in the *ascB* region. Clones were screened without an enrichment to avoid off- target mutations. The efficiency of insertion of the 10-*nt* was 5%.

### Plasmid construction

For promoter assays, the *asc* operon was cloned from the end of *ascB* gene to the *ascF* gene in increments of 1000 bp into the *EcoR*I and *Kpn*I site of pProbe-NT' plasmid and analyzed for the expression of *gfpuv*. Clone A5 encoding the entire of *ascB* gene and a truncated *ascF* was verified to express *gfpuv*.

Strains harboring the plasmid A5 or pProbe-NT' were cultured overnight in LB medium supplemented with kanamycin. Then, 1% (1:100) of overnight culture was transferred to fresh LB and grown to an OD of 0.7. Next, 180 μL of cells were transferred to a 96-well plate and 2 g/L of glucose or cellobiose was added. Cell growth and GFP expression were monitored every 10 min by measuring OD_600_ and fluorescence emission at 535 nm (excitation at 475 nm), respectively, using the Tecan SpectraFluor Plus plate reader (Tecan-US, Durham, NC). Relative fluorescence was calculated by normalizing the GFP values for the corresponding values of OD_600_.

### Multiplex Genome Engineering

Oligonucleotides were designed to randomize the RBS regions of *chbB*, *chbA*, *chbC*, *chbF*, *ascF* and *ascB* genes and the promoter regions of *chb* and *asc* operon. OSS strains were transformed with the plasmid pSIM5. The RBS location was predicted using the RBSDesigner program [8]. Multiplex genome engineering was performed as described elsewhere but with a simple modification [9]. Seven oligos were mixed at a concentration of 0.3 μM per oligo. Following every two cycles of genome engineering, strains were enriched on cellobiose to an OD_600_ of 0.5 and the enriched population was subjected to a further MAGE cycle. The MAGE cycles were repeated eight times with enrichment on cellobiose between two MAGE cycles. Approximately 282 clones were scored for efficient growth on cellobiose in 2-mL volume 96-well plates. The top scoring strains were analyzed again in test tubes for efficient growth on cellobiose. The entire *chb* and *asc* operons of the efficient cellobiose metabolizing strain were sequenced to identify the RBS regions mutated through genome engineering.

### qRT-PCR and 5' RACE

Total RNA was isolated (using Qiagen RNA kit) from mid-log phase culture (OD of 0.3–0.4) of OSS, OSS-yebK*, OSS-ascB*, and ESS grown on cellobiose minimal medium. MG1655 grown on glucose minimal medium was used as a control. For qRT-PCR, 0.5 μg of total RNA was used to synthesize cDNA using M-MuLV reverse transcriptase and appropriate dilutions of cDNA were used as templates for qRT-PCR. The qRT-PCR was performed as described previously using the 2X SYBR Green master mix [10]. Different dilutions of genomic DNA were used as a standard for comparison. All samples were normalized to the 16S rRNA level.

5' RACE PCR was performed as described previously [11]. The resulting PCR product was sequenced at Macrogen, Inc. using the *ascF*-5'-GSP2 and *ascB*-5'-GSP2 primers respectively for *ascF* and *ascB* RACE.

### YebK purification and EMSA

YebK or YebK* was expressed with 6His tags from pET31b+ plasmid. Proteins were purified as described before but with a simple modification [12]. In the last step, the purified proteins were maintained in Tris buffer as HEPES buffer led to the immediate precipitation of the protein. The template for EMSA was PCR amplified from MG1655 genomic DNA using the primer pairs: YebK_EMSA_FP and YebK_EMSA_RP. Next, 150 ng of the DNA was incubated with proteins in a concentration ranging from 0 to 1 μM in a binding buffer (16 mM Tris (pH 7.5), 3 mM MgCl_2_, 30 mM NaCl, 0.0065% Triton-X and 0.033 mg/mL BSA) for 1 hour at 37°C. When indicated, keto-deoxy-6-phosphogluconic acid (KDPG) was substituted at a final concentration of 3 mM. The reaction was analyzed on 5% non-denaturing polyacrylamide gel for YebK or 7% non-denaturing polyacrylamide gel for YebK*.

## Results

### Mutations pertaining to cellobiose metabolism in strain ESS

**Fig 1:**
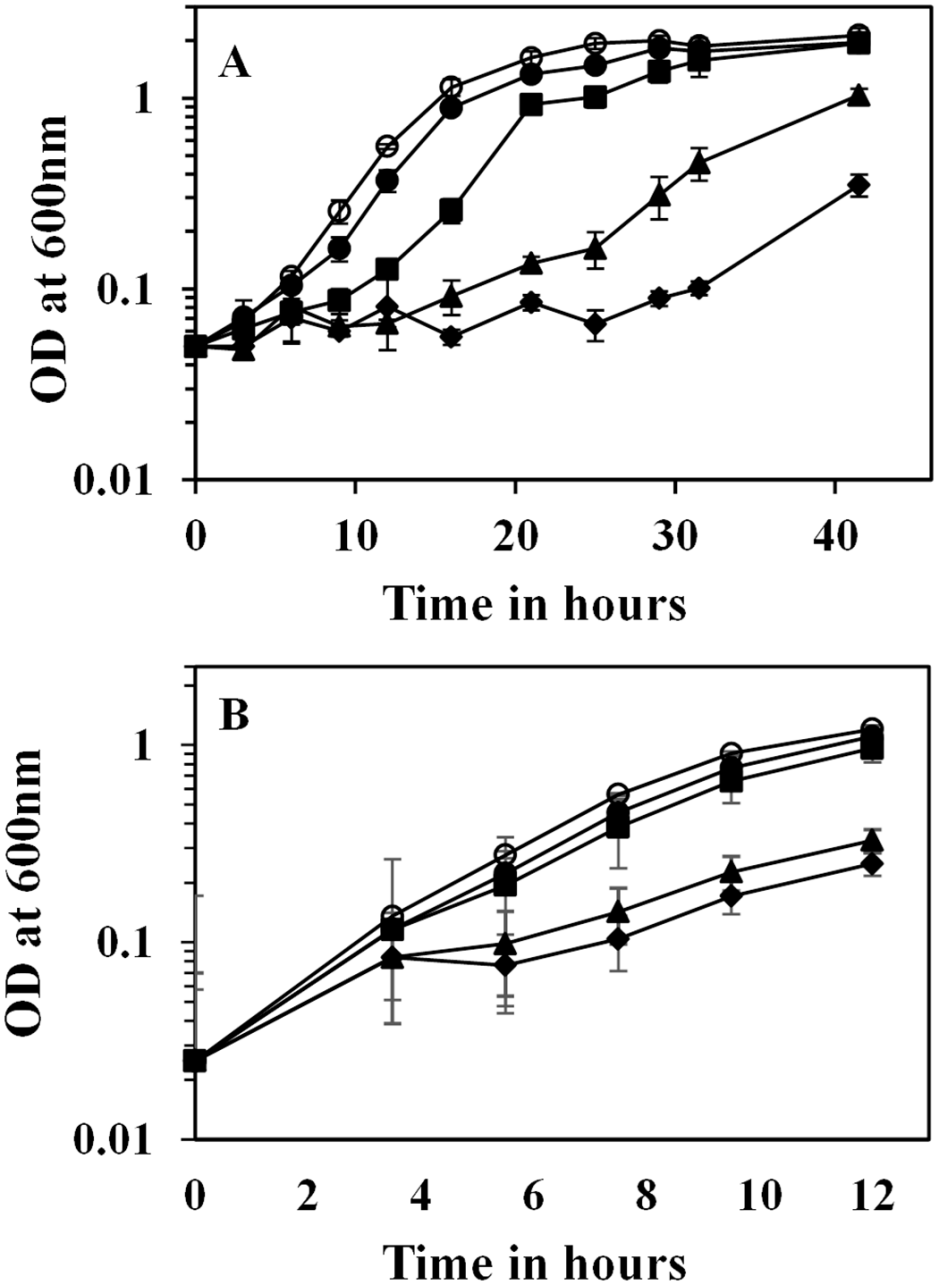
(A) Comparison of growth of *E*. *coli* strains on cellobiose minimal medium. For these experiments, strains were grown overnight in LB, washed with M9 salts, and suspended in fresh M9-cellobiose minimal medium to a final OD of 0.05. OSS–*closed diamond*; OSS-yebK*–*closed triangle*; OSS-ascB*–*closed square*; OSS-yebK*/ascB*–*closed circle*; ESS–*open circle*. Error bars indicate the standard deviation of experiments performed in triplicate. (B) Comparison of growth of *E*. *coli* strains on cellobiose minimal medium. These strains were pre-adapted on M9-cellobiose minimal medium to mid-log phase and diluted into fresh M9-cellobiose minimal medium. OSS–*closed diamond*; OSS-yebK*–*closed triangle*; OSS-ascB*–*closed square*; OSS-yebK*/ascB*–*closed circle*; ESS–*open circle*. Error bars indicate the standard deviation of experiments performed in triplicate.

ESS is one of the efficient cellobiose metabolizing strains of *E*. *coli* constructed by replacing the cryptic promoters of *chb* and *asc* operons with constitutive promoters and adaptive evolution on cellobiose [6]. Comparative whole genome re-sequencing revealed 12 and 16 mutations in strains OSS and ESS, respectively. Comparison of the mutations in ESS and OSS with the reference sequence of MG1655 indicates that at least 11 mutations differed between the reference strain of MG1655 and the laboratory strain. Conventional PCR-based re-sequencing helped to verify only two true-positive mutations in strain ESS, yet through this approach longer deletion and insertion could not be identified. The first mutation leads to the duplication of 10-*nt* (GAGGATGAAA) upstream of the *ascB* gene of the *asc* operon. The second mutation was a nonsense mutation on a previously uncharacterized transcription factor, *yebK*, resulting in the expression of a truncated protein (79 amino acids) containing only the DNA binding domain. We then characterized the role of independent mutations on cellobiose metabolism. Allelic replacement of each of the two mutations independently or in combination in strain OSS leads to enhancing the growth rate on cellobiose in the following order: OSS-yebK*ascB*>ascB*>yebK*>OSS, indicating that both these mutations are beneficial for growth on cellobiose (Fig 1A and 1B). The final strain, OSS-yebK*ascB*, had a specific growth rate similar to the strain ESS when grown on cellobiose minimal medium, indicating that key mutations related to cellobiose metabolism in strain ESS was deciphered.

### Role of AscB* in cellobiose metabolism

**Fig 2:**
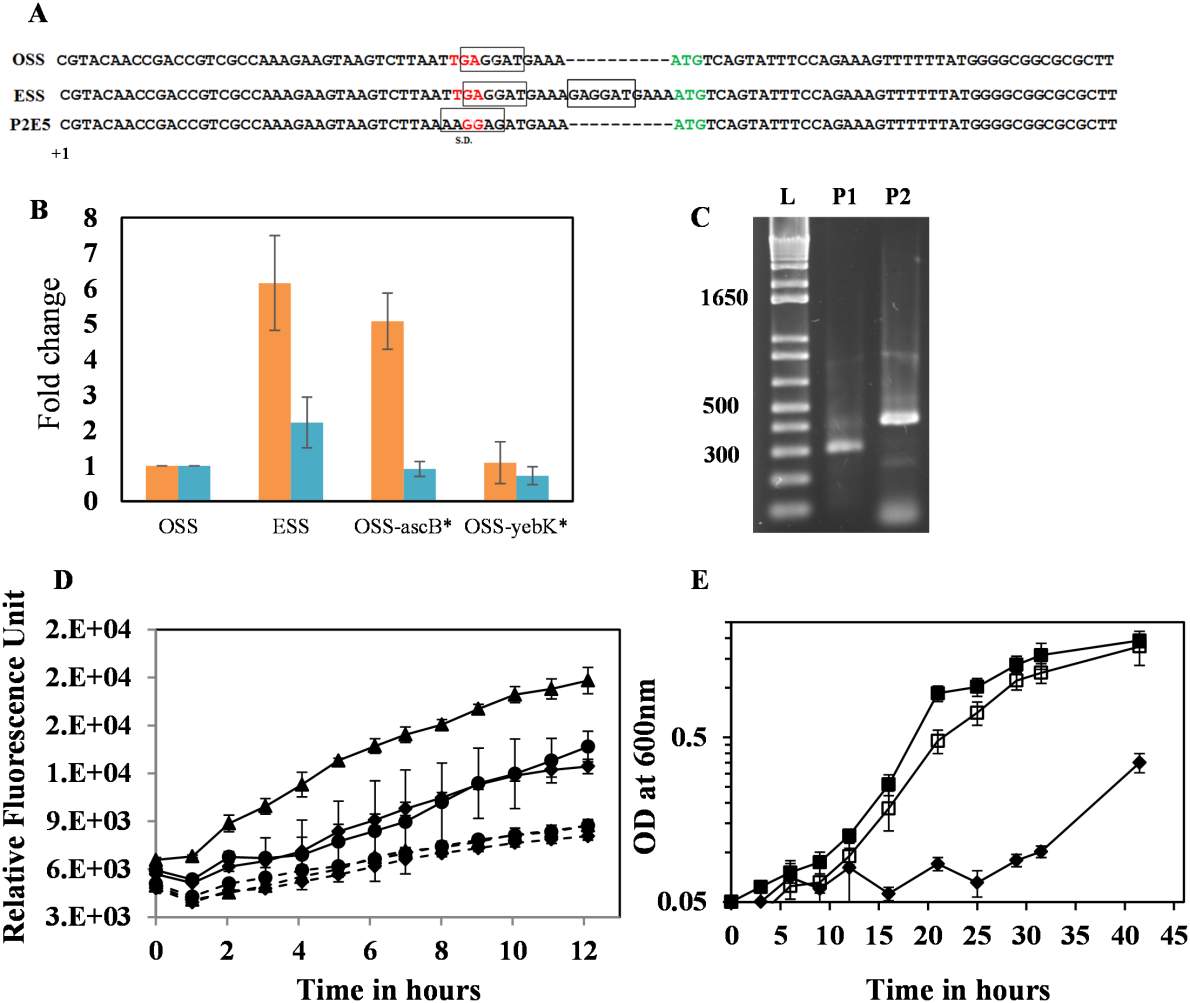
(A) Figure indicating the predicted RBS of OSS and the duplicated sequence observed in strain ESS. The two putative RBS of strain ESS are shown in boxes. The figure also indicates the mutated RBS in strain P2E5 obtained through genome engineering. The new transcription start site of *ascB* gene was marked as +1. (B) Fold change in the expression level of *ascB* (*orange*) or *ascF* (*blue*) in ESS, OSS-yebK*, and OSS-ascB* relative to the expression of the corresponding genes in strain OSS. Error bars indicate the standard deviation of experiments performed in triplicate. (C) 5' RACE PCR to map the location of TSS for *ascF* and *ascB*. P1 corresponds to the transcript amplifiable by *ascF*_RACE_RP and is expected to be around 262 bp; P2 corresponds to the transcript amplifiable by *ascB*_RACE_RP and is expected to be around 1995 bp. The RACE PCR product of *ascF* corresponds to the expected size of P1, whereas the RACE PCR product of *ascB* was smaller than the expected size corresponding to the P2 transcript, indicating the possibility of new TSS. (D) Assay of putative promoter within the *asc* operon. The sequences from +391 from the start codon of *ascF* to the end of the operon were cloned into the *Eco*RI and *Kpn*I site of pProbe-NT' vector. *Solid lines*–pProbe-A5; *dotted lines*–pProbe-NT'; *solid circle*–MG1655; *solid diamond*–MG1655-ΔP_*ascG*_-*ascB*::*frt*, and *solid triangle*–MG1655-Δ*chbB*-*chbF*::*frt*. (E) Comparison of growth on cellobiose. OSS–*closed diamond*; OSS-ascB*–*closed square*; OSS-P2E5 –*open square*. Error bars indicate the standard deviation of experiments performed in triplicate.

The 10-*nt* sequence upstream of the start codon of *ascB* gene was duplicated in the strain ESS. The duplicated nucleotide would likely be related to enhancing the translational or transcriptional efficiency of the *ascB* gene. Prediction of the RBS location of *ascB* in strain ESS indicates that the strain carries a tandem RBS sequence (Fig 2A and S1 Fig). We previously reported through transcriptomic analysis that the *ascB* expression level was 5-fold higher in ESS than in OSS [13]. We then analyzed if the increase in *ascB* expression level is a consequence of mutations in *yebK* or *ascB*. Strains carrying the duplication of 10-*nt* upstream of *ascB* start codon show a significant increase in *ascB* expression level relative to strain OSS (between 5- and 6-fold) (Fig 2B).

However, the gene *ascB* is located at the 3' end of the *asc* operon and there is no significant up-regulation of *ascF* in different strains such as OSS, OSS-ascB*, or OSS-yebK* (Fig 2B). Hence, we hypothesized that *ascB* is transcribed independent of the *ascF* gene. It is possible that there is an additional promoter within the *asc* operon regulating the *ascB* gene. Hence, using 5' RACE, we mapped the 5' region from *ascF* or *ascB* mRNA. The length of the 5' RACE PCR product was 262 bp as expected and the Transcription Start Site (TSS) of *ascF* mRNA was mapped to the TSS introduced along with the constitutive promoter (Fig 2C). However, the size of the 5' RACE PCR from *ascB* was 500 bp, which is shorter than the expected 1995 bp for a full-length *ascFB* transcript (Fig 2C). Upon sequencing the RACE PCR product, the TSS was mapped to 1419 bp from the *ascF* start codon and was approximately 38 bp upstream of the mutation observed in strain ESS. Promoter assay indicates the presence of a putative promoter in the middle of the *ascF* gene (Fig 2D). In addition, the putative promoter activity was enhanced in MG1655/ΔP_*asc*_-*ascB*::frt strains grown on LB medium supplemented with glucose relative to that in strains MG1655 or MG1655/Δ*chbB*-*chbF*::frt. This result indicates that the putative internal promoter is regulated by the *ascG* gene product of the *asc* operon. These results indicate that the modifications (10-*nt* insertion leading to the duplication of RBS sequence) observed in the intergenic region of *ascB* in ESS would play a potential role in modulating the transcriptional or translational efficiency in the 5' untranslated region (UTR) of the *ascB* transcript.

### Optimizing the cellobiose metabolic pathway through oligo-mediated recombineering

To further verify if only *ascB* is rate-limiting for cellobiose metabolism or whether other genes related to *chb* and *asc* operons could enhance cellobiose metabolism, two constitutive promoters (of the *chb* and the *asc* operons) and RBS of six genes (*chbB*, *chbC*, *chbA*, *chbF*, *ascF*, and *ascB*) were randomized through oligo-mediated genome engineering. Consistent with the genotype observed in the strain ESS, efficient cellobiose metabolizing strains obtained through genome engineering had mutations in the upstream region of the *ascB* gene, leading to a change in the spacer length (Fig 2A). One representative mutant, OSS-P2E5 (Fig 2E), had a growth rate similar to strain OSS-ascB*. These results indicate that the *ascB* gene indeed might have a significant role in cellobiose metabolism beyond being present in a minor/incomplete operon.

### Role of YebK in cellobiose metabolism

**Fig 3:**
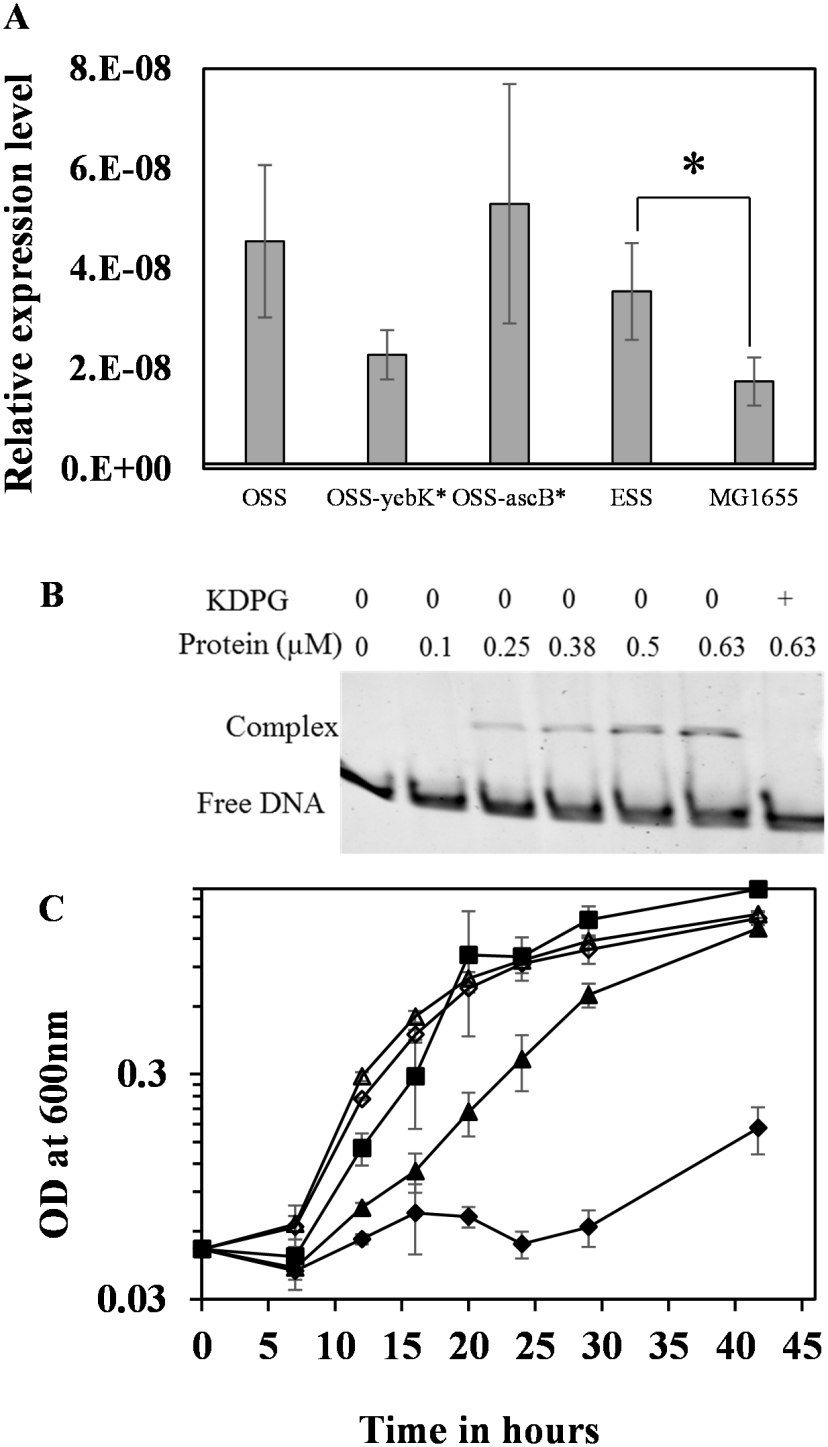
(A) Comparison of the relative level of *yebK* mRNA in strains OSS, OSS-yebK*, OSS-ascB*, and ESS grown on cellobiose minimal medium. MG1655 RNA was prepared from cells grown on glucose minimal medium. Error bars indicate the standard deviation of experiments performed in triplicate. Statistical analysis was performed using one-way ANOVA with MG1655 grown on glucose minimal medium as control. Asterisk indicates the statistically significant comparison with p<0.05. The p value was marked for one indicative strain grown on cellobiose compared to the wild type grown on glucose. Statistical analysis indicates that difference in *yebK* expression-level was significant between glucose and cellobiose grown cells but not among different strains growing on cellobiose. (B) EMSA to analyze the auto-regulatory role of 6 His-YebK. The promoter of *yebK* was incubated with different concentration of purified 6 His-YebK protein in the presence or absence of KDPG and analyzed on 5% non-denaturing polyacrylamide gel. (C) Growth of *edd* gene deleted strains on cellobiose minimal medium. OSS—*closed diamond*; OSS-yebK*–*closed triangle*; OSS-Δ*eda*–*open diamond*; OSS-yebK*/Δ*eda*–*open triangle*; OSS-ascB*–*closed square*. Error bars indicate the standard deviation of experiments performed in triplicate.

The second mutation observed in ESS, *yebK*, was found to have a dominant role only upon transfer from rich medium to minimal cellobiose medium (Fig 1A and 1B and S1 Table). Cells pre-cultured on cellobiose minimal medium exhibited negligible impact with respect to the presence or absence of *yebK* mutation, indicating its predominance only when transferred from a rich medium to a minimal medium (Fig 1B and S1 Table). The expression level of *yebK* was higher in strains growing on cellobiose than in wild type strain grown on glucose minimal medium indicating its predominance in cellobiose-minimal medium (Fig 3A).

Mutations in *yebK* help in reducing the length of lag phase for growth with cellobiose as a sole carbon source when introduced independently into strains OSS or OSS-ascB*. Several factors are proposed to have an influence on the lag phase, including the age and size of the initial inoculum, and the physicochemical composition of the new medium, that is, cellobiose [14]. Since all strains used in this study express the cellobiose metabolic pathway constitutively, the time required to activate cellobiose metabolism may not be the major reason for the lag phase in cellobiose medium. Cell viability was maintained constant for up to 20 hours in LB medium for different strains with and without the *yebK* mutation (S2A Fig). Hence, difference in the lag phase upon shift from a rich medium to an M9-cellobiose minimal medium (between strains with wild type and mutant *yebK*) is not a consequence of difference in cell viability of the initial inoculum. Similarly, viability of strains OSS or OSS-yebK* was maintained constant throughout the long lag phase, indicating that the lag phase is not due to toxic or osmotic effects of cellobiose (S2B Fig).

### YebK recognizes the central metabolic intermediates as co-factor

Previously, it was reported that YebK orthologs of *Pseudomonas* and *Shewanella* species regulate the Entner–Doudoroff (ED) pathway and gluconeogenesis of the central metabolic pathway respectively, using KDPG as an effector molecule [15]; it is possible that YebK could also recognize similar effector molecules and regulate the central metabolic pathway. We hypothesized that the lag phase observed in cellobiose minimal medium in strains expressing wild type *yebK* could be because of the requisite to efficiently modulate the central metabolic pathway. The target genes regulated by YebK are not known; however, it is reported through a comparative genomic reconstruction that the HexR family of proteins are autoregulatory [15], and hence we used the *yebK* gene’s own promoter in Electrophoretic Mobility Shift Assay (EMSA) to determine if YebK could also recognize the central carbon intermediates, such as KDPG, as a co-factor. As shown in Fig 3B, the 6His-YebK binds to its own promoter and the binding is reversed in the presence of KDPG. Interestingly, the truncated YebK (6 His-yebK*) also retained the DNA binding ability (S3A Fig).

### YebK inactivation is important for controlling lag phase in cellobiose metabolism

To further verify whether disrupting the DNA binding ability of YebK is essential to counteract the lag phase observed upon transfer from rich medium to cellobiose minimal medium, the *eda* gene encoding the ED pathway enzyme, KDPG aldolase, was deleted in OSS and its growth characteristic on cellobiose was analyzed. Deletion of the *eda* gene would result in intracellular accumulation of KDPG, which in turn could help in reversing the DNA binding ability of YebK. Similar to that observed in OSS-yebK*, the lag phase was reduced in OSS-Δ*eda* compared to strain OSS (Fig 3C). These results provide evidence that the lag phase could be reversed by inactivating or impairing the DNA-binding ability of YebK.

Deletion of the *edd* gene, encoding the first enzyme of the ED pathway (phosphogluconate dehydratase), results in no production of KDPG and hence the resulting strain OSS-Δ*edd* could not grow on cellobiose minimal medium even after 96 hours (3 days) of cultivation, indicating the need for KDPG to inactivate *yebK* before starting to grow on cellobiose (S3B Fig). Expressing yebK* in OSS-Δ*edd* results in the same phenotype as that observed with OSS-yebK*, thus signifying that deregulation of the ED pathway is not the ultimate effect of *yebK* inactivation and there could be other pathways that were controlled by YebK using KDPG as one of the signal effectors. Further characterization of genes directly regulated by YebK is essential to establish the clear regulatory events controlled by YebK.

## Discussion

In this study, we report the molecular characterization of independent mutations found in strain ESS in order to decipher the genetic events that helped in enhancing the cellobiose metabolism in *E*. *coli*. Strain OSS expresses two different phospho-β-glucosidases: ChbF and AscB. However, it was intriguing whether the AscB protein from the *asc* operon (and not ChbF) plays a significant role in enhancing cellobiose metabolic ability in strain ESS and in P2E5. Previous reports indicate that even with high selection stringency on cellobiose, the *asc* operon could not support growth with cellobiose as a sole carbon source [3]. We have previously expressed the *asc* operon under a constitutive promoter and the strains could still not grow on cellobiose [6]. Thus, it could be possible that the potential of AscB for cellobiose was under-estimated by the synergistically acting transporter protein, AscF. The importance of *ascB* for cellobiose metabolism (as described in this study) could be a major reason for the conservation of the cryptic operon through the evolution. In accordance with these findings, the *asc* operon orthologs evolved to retain the *ascB* gene in different lineages of *Enterobacter* species. Furthermore, the new TSS identified within the *asc* operon indicate that the TSS predicted within the intergenic or coding regions of the gene are also essential regulatory nodes and could serve as a potential target for metabolic engineering and strain optimization. Recent advances in high-throughput screening techniques have reported the presence of such additional TSS within an operon [16].

While there are several pioneering studies on the transcriptional regulation during the stationary phase mediated by *rpoS* and related genes, it is relatively challenging to study the response of transcription factors controlling the lag phase or transition from one environmental condition to the other. Lag phase is a poorly described phase in bacterial growth stages [14]. Even with *E*. *coli* (well-studied microorganism), about 40% of the genes are uncharacterized [17], mainly because there is no prior knowledge of the physiological conditions where the gene-of-interest plays a dominant role. Several high-throughput screening tools, including Phenotype Microarray [17], and metabolite profiling [18], were used for the functional assessment of the uncharacterized genes. It is still difficult to characterize the function of putative transcription factors, because (except for a few regulators) most transcription regulators would affect the lag phase or specific growth rate or cause flux rerouting without any significant phenotypic changes [19]. In this study we demonstrate that the transcription factor YebK helps in functionally coupling the minimal nutrient condition to the central carbon metabolism by modulating the length of lag phase relative to the specific growth rate of the strain. There could be several speculations on the demand for such modulations in the central carbon metabolism, including redox balance, maintenance of particular level of signaling metabolites, and increasing the energy efficiency. Further studies on the target genes regulated by YebK would help in understanding the regulatory changes put forth by YebK upon transfer from a rich medium to a minimal medium.

Several studies were performed in *E*. *coli* strains adapted on known carbon sources, including lactate, acetate, glucose, or glycerol [20,21]. Interestingly, adaptive evolution on native (but poorly metabolized) carbon sources (such as lactate or glycerol) resulted in mutations in stress-related regulatory genes (like *rpoS*, *hfq*) [22], global regulators (like *cyaA*, *crp*) [22], or housekeeping genes (like *rpoC*) [20], whereas adaptation on a non-native carbon source did not have any mutation in such global regulatory genes. Instead, this study provides new insights, while engineering *E*. *coli*, for growth on cellobiose, though these mechanisms are restricted to the PTS-mediated cellobiose metabolism.

To our knowledge, this study is the first to report the conditions under which the transcription factor *yebK* exhibits its impact on *E*. *coli* growth. The global transcription regulators reported so far regulate a specific nutrient condition. For instance, *crp* is a global carbon regulator, *arcA* is a regulator of anoxic conditions, and *narL* is a nitrate/nitrite responsive regulator. Similarly, *yebK* could serve as a global regulator, controlling the shift in nutrient conditions from a rich medium to a minimal medium. Further studies are needed to explore in depth the molecular mechanisms of such regulation mediated by *yebK*.

## Supporting Information

S1 Fig Nucleotide sequences of *ascFB* operon in strain ESS. The scar sequence and the CP12 promoter is indicated in blue; the TSS of *ascF* and *ascB* are indicated in red and marked as +1; the nucleotide sequences of *ascF* and *ascB* are indicated in green and dark blue respectively; the duplicated nucleotide above *ascB* gene in strain ESS is indicated in purple; the RBS sequence of *ascF* and *ascB* of strain ESS is enclosed in a box; the native RBS of *ascB* is indicated as a dotted box.
(TIF)

S2 Fig (A) Comparison of growth on LB: OSS–*closed diamond*; OSS-yebK*–*closed triangle*; OSS-ascB*–*closed square*; OSS-yebK*/ascB*–*closed circle*; ESS–*open circle*. Samples were collected at the indicated time, diluted serially, and plated on LB-agar medium. Colonies were counted after 12 hours of plating (B) Comparison of cell viability during the lag phase in OSS (closed diamond) and OSS-yebK* (closed triangle) growing on cellobiose minimal medium. Samples were collected at the indicated time, diluted serially, and plated on LB-agar medium. Colonies were counted after 12 hours of plating.
(TIF)

S3 Fig (A) EMSA for 6His-YebK* protein. The promoter of *yebK* was incubated with different concentration of purified 6His-YebK* protein in the presence or absence of KDPG and analyzed on 7% non-denaturing polyacrylamide gel. (B) Growth of *edd* gene deleted strains on cellobiose minimal medium. OSS—closed diamond; OSS-yebK*–closed triangle; OSS-Δ*edd*–open diamond; OSS-yebK*/Δ*edd*–open triangle.
(TIF)

S1 Table Specific growth rate of different strains growing on cellobiose-minimal medium. (DOCX)
