# Correction: Novel Functions and Regulation of Cryptic Cellobiose Operons in *Escherichia coli*

**DOI:** 10.1371/journal.pone.0143463

**Published:** 2015-11-16

**Authors:** Vinuselvi Parisutham, Sung Kuk Lee

In the Funding section, a grant number from the funder Next-Generation Bio Green 21 Program is listed incorrectly. The correct grant numbers are: SSAC, PJ011181. The complete, correct funding statement is:

This work was supported by the National Research Foundation of Korea (NRF) through grants funded by the Ministry of Science, ICT & Future Planning (MSIP) (NRF-2009-C1AAA001-2009-0093491), by the Intelligent Synthetic Biology Center of Global Frontier Project funded by the MSIP (2011–0031948), and by a grant from the Next-Generation Bio Green 21 Program (SSAC, PJ011181), Rural Development Administration, Republic of Korea.
