# Supplementary figures and images for: Novel Functions and Regulation of Cryptic Cellobiose Operons in Escherichia coli
Source: PLoS One. 2015 Jun 29;10(6):e0131928. doi: 10.1371/journal.pone.0131928 (PMC4488073; doi:10.1371/journal.pone.0131928)

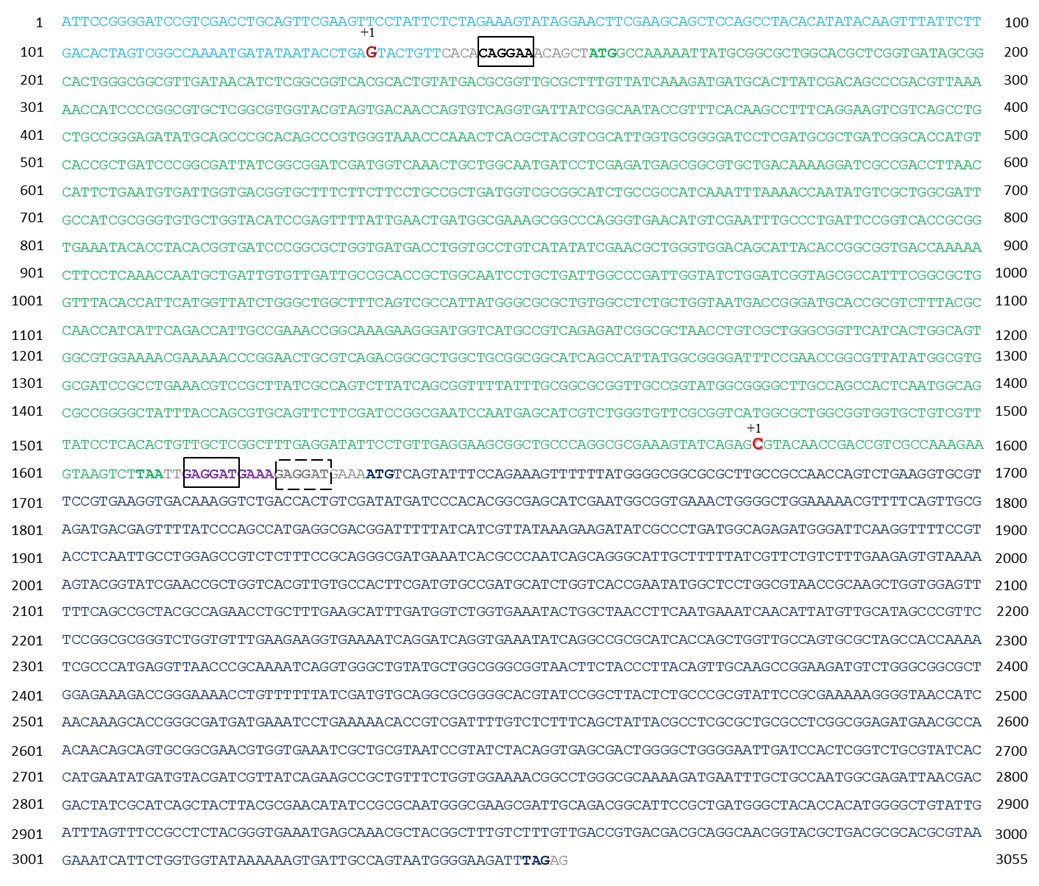

Supplement: S1 Fig — The scar sequence and the CP12 promoter is indicated in blue; the TSS of ascF and ascB are indicated in red and marked as +1; the nucleotide sequences of ascF and ascB are indicated in green and dark blue respectively; the duplicated nucleotide above ascB gene in strain ESS is indicated in purple; the RBS sequence of ascF and ascB of strain ESS is enclosed in a box; the native RBS of ascB is indicated as a dotted box. (TIF) [file pone.0131928.s001.tif]

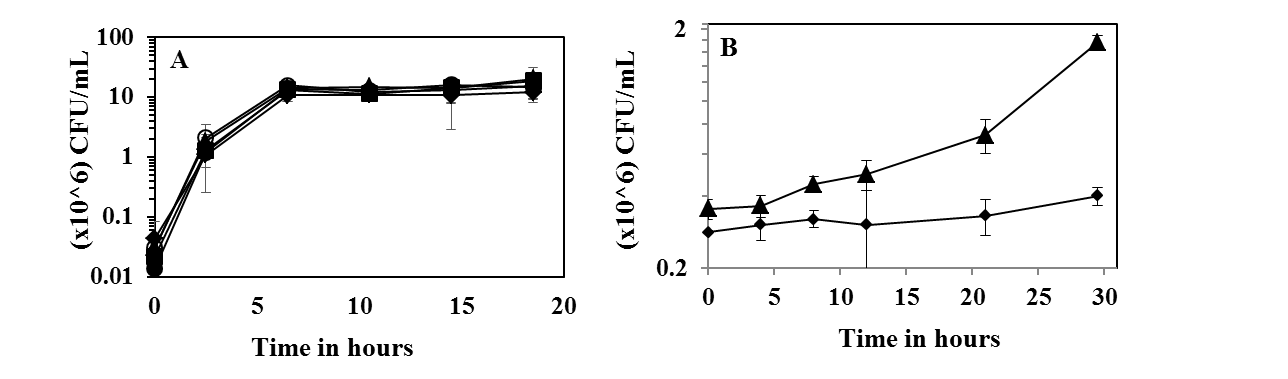

Supplement: S2 Fig — (A) Comparison of growth on LB: OSS–closed diamond; OSS-yebK*–closed triangle; OSS-ascB*–closed square; OSS-yebK*/ascB*–closed circle; ESS–open circle. Samples were collected at the indicated time, diluted serially, and plated on LB-agar medium. Colonies were counted after 12 hours of plating (B) Comparison of cell viability during the lag phase in OSS (closed diamond) and OSS-yebK* (closed triangle) growing on cellobiose minimal medium. Samples were collected at the indicated time, diluted serially, and plated on LB-agar medium. Colonies were counted after 12 hours of plating. (TIF) [file pone.0131928.s002.tif]

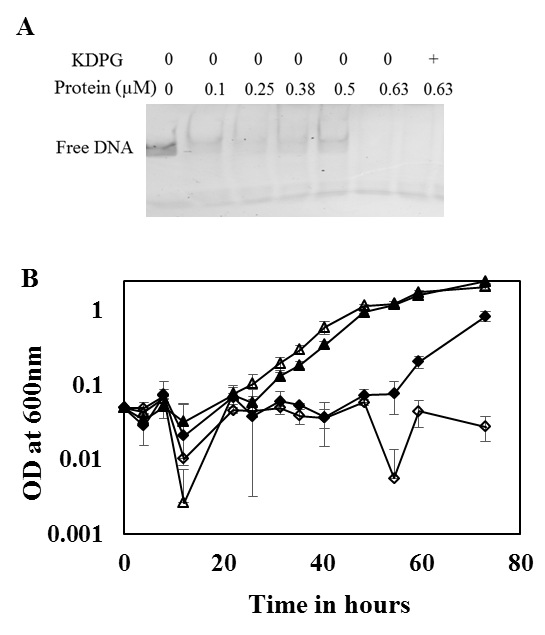

Supplement: S3 Fig — (A) EMSA for 6His-YebK* protein. The promoter of yebK was incubated with different concentration of purified 6His-YebK* protein in the presence or absence of KDPG and analyzed on 7% non-denaturing polyacrylamide gel. (B) Growth of edd gene deleted strains on cellobiose minimal medium. OSS—closed diamond; OSS-yebK*–closed triangle; OSS-Δedd–open diamond; OSS-yebK*/Δedd–open triangle. (TIF) [file pone.0131928.s003.tif]
