# Supplementary material for: Novel Functions and Regulation of Cryptic Cellobiose Operons in Escherichia coli
Source: PLoS One. 2015 Jun 29;10(6):e0131928. doi: 10.1371/journal.pone.0131928 (PMC4488073; doi:10.1371/journal.pone.0131928)
Supplement: S1 Table — (DOCX) [file pone.0131928.s004.docx]

**Supp. Table 1.** Specific growth rate of different strains on cellobiose-minimal medium. Slope of the semi-logarthamic graph was calculated using the LINEST function. Data represent the average (± SD) of three independent replicates.

|  | Directly transferred from LB to M9-cellobiose minimal medium (hr^-1^) | Pre-adapted in M9-cellobiose minimal medium (hr^-1^) |
| --- | --- | --- |
| OSS | 0.034 ± 0.0060 | 0.180 ± 0.0090 |
| ESS | 0.100 ± 0.0020 | 0.330 ± 0.0026 |
| OSS-*yebK** | 0.073 ± 0.0070 | 0.210 ± 0.0059 |
| OSS-*ascB** | 0.107 ± 0.0040 | 0.310 ± 0.0029 |
| OSS-*yebK**/*ascB** | 0.101 ± 0.0030 | 0.320 ± 0.0041 |
